# FANCA deficiency promotes leukaemic progression by allowing the emergence of cells carrying oncogenic driver mutations

**DOI:** 10.1038/s41388-023-02800-9

**Published:** 2023-08-12

**Authors:** Patrycja Pawlikowska, Laure Delestré, Sebastian Gregoricchio, Alessia Oppezzo, Michela Esposito, M’ Boyba Diop, Filippo Rosselli, Christel Guillouf

**Affiliations:** 1 CNRS UMR9019, Université Paris-Saclay, Gustave Roussy Cancer Campus, Villejuif, France; 2 Equipe Labellisée Ligue Nationale Contre le Cancer, Villejuif, France; 3 Inserm UMR1170, Université Paris-Saclay, Gustave Roussy Cancer Campus, Villejuif, France; 4 Present Address: Inserm U981, Gustave Roussy Cancer Campus, CNRS UMS3655, Inserm US23AMMICA, Villejuif, France; 5 Present Address: Division of Oncogenomics, Oncode Institute, The Netherlands Cancer Institute, Amsterdam, The Netherlands

**Keywords:** Haematological cancer, DNA damage and repair

## Abstract

Leukaemia is caused by the clonal evolution of a cell that accumulates mutations/genomic rearrangements, allowing unrestrained cell growth. However, recent identification of leukaemic mutations in the blood cells of healthy individuals revealed that additional events are required to expand the mutated clones for overt leukaemia. Here, we assessed the functional consequences of deleting the Fanconi anaemia A (*Fanca*) gene, which encodes a DNA damage response protein, in *Spi1* transgenic mice that develop preleukaemic syndrome. FANCA loss increases SPI1-associated disease penetrance and leukaemic progression without increasing the global mutation load of leukaemic clones. However, a high frequency of leukaemic FANCA-depleted cells display heterozygous activating mutations in known oncogenes, such as *Kit* or *Nras*, also identified but at low frequency in FANCA-WT mice with preleukaemic syndrome, indicating that FANCA counteracts the emergence of oncogene mutated leukaemic cells. A unique transcriptional signature is associated with the leukaemic status of FANCA-depleted cells, leading to activation of MDM4, NOTCH and Wnt/β-catenin pathways. We show that NOTCH signalling improves the proliferation capacity of FANCA-deficient leukaemic cells. Collectively, our observations indicate that loss of the FANC pathway, known to control genetic instability, fosters the expansion of leukaemic cells carrying oncogenic mutations rather than mutation formation. FANCA loss may contribute to this leukaemogenic progression by reprogramming transcriptomic landscape of the cells.

## Introduction

Genetic instability, a key characteristic of cancer [1], is considered a major cell autonomous force in both cancer initiation and progression. Indeed, an increased mutational landscape correlates with cancer progression, as supported by three major facts: (a) the majority of solid tumours and leukaemia consistently exhibit genetic instability, as revealed by both cytogenetic and sequencing data analyses [2]; (b) the high risk of cancer incurred by patients bearing inactivating germline mutations in genes encoding DNA damage response (DDR) proteins, which maintain genome stability [3]; and, inversely, (c) the frequent presence of inactivating mutations in DDR protein-encoding genes in sporadic tumours [4].

However, the mutational signature repertoire and the mutational load associated with cancer are clearly tumour-dependent and, comparatively to solid tumours, scarce in leukaemia [2]. Moreover, recently published studies revealed an accumulation of somatic mutations in cancer-associated driver genes in healthy cells that maintain their physiological functions [5]. For example, a pattern of somatic mutations found in haematological cancer-associated genes, including *DNMT3A*, *TET2*, *ASXL1*, *TP53* and its regulator *PPM1D*, has been described in healthy individuals without clinical haematologic abnormalities [6]. These observations raised questions about what additional factors and events, in addition to genetic instability, contribute to leukaemic progression.

SPI1/PU.1 is a master TF involved in normal and stressed haematopoiesis. SPI1 behaves either as a tumour suppressor [7, 8] or an oncogene [9–11]. Alteration of SPI1 expression in a mouse model has been identified as a founding event in myeloid transformation, triggering cellular and molecular alterations that are consistent with preleukaemic disorders [12–14]. SPI1-overexpressing mice develop such a preleukemic disorder from erythroid origin [14]. SPI1 constitutive expression in mice causes haematological neoplasia characterized by anaemia due to the blockage of erythroid differentiation and splenomegaly due to expansion of blasts whose expansion is still controlled by growth factors [14]. Whereas 60% of mice develop preleukaemic syndrome, they rarely progress spontaneously into leukaemia (identified in less than 10% of the offspring). However, leukaemic progression can be mediated by additional mutations in the *c-Kit* gene encoding the stem cell factor (SCF) receptor [15]. Thus, transgenic *Spi1*-overexpressing (Tg*Spi1*) mice represent a preleukaemic state towards the development of leukaemia. Similarly, a block of erythroid differentiation due to high expression of transcriptional regulators has recently been described in over 25% of acute human erythroleukaemia (AEL) cases [16]. The identification of secondary mutations in genes encoding DDR proteins in murine models of AEL [17], including those that code for the proteins of the Fanconi anaemia pathway (FANC), supports that downstream alteration in key oncogenes and abnormalities in DDR plays a role in leukaemic progression.

FANC proteins constitute a pathway embedded in the DDR network and are involved in DNA repair and replication rescue, ensuring chromosomal integrity [18, 19]. Their genetic loss of function leads to Fanconi anaemia (FA), a human bone marrow failure (BMF) syndrome associated with DDR failure, genetic instability, abnormalities in ribosome biogenesis and a proinflammatory and prosenescent phenotype [20, 21]. Counterintuitively, considering hypoproliferative FA cells and clinical phenotypes, FA patients are at high risk for myelodysplastic syndrome (MDS), acute myeloid leukaemia (AML) and solid cancers [22]. FA is due to inactivating biallelic mutations in over 20 *FANC* genes, some of which have also been involved in other cancer predisposition syndromes [23, 24]. Surprisingly, patients with mutations in the different genes that affect the functionality of the FANC pathway do not have the same spectrum of cancers [23, 25, 26]. Moreover, FANC-knockout (KO) mouse models generally fail to spontaneously develop leukaemia or other cancers, but they present minor BMF defects, infertility and DNA damage hypersensitivity, attesting to the presence of major DDR alterations [27–30]. Thus, even if myelodysplasia or AML in FA patients appears to be associated with some specific genomic abnormalities [31], genetic instability seems to be insufficient per se for overt leukaemic development in FA.

To provide new insights into additional factors or events that, in addition to genetic instability, contribute to leukaemic progression, we combined alternative genetic preleukaemic settings provided by the SPI1-overexpressing mice and the knockout of the *Fanca* gene. Since preleukaemic erythroblastic cells expressing high levels of SPI1 exhibit replicative stress [32, 33], a deficiency in DNA repair and/or replicative stress responding proteins could increase the genetic instability and/or tumorigenic progression of SPI1-overexpressing preleukaemic blasts, revealing gene products and pathways involved in allowing leukaemia-initiated cells to progress. We show that loss of function of FANCA and abnormally high expression of SPI1 synergize during leukaemogenesis. Depletion of FANCA in TgSpi1 mice leads to progression from preleukaemia to leukaemia without increasing mutation load. In contrast, selection and/or expansion of cells with oncogenic mutations are promoted and associated with a peculiar transcriptional programme in leukaemic cells.

## Results

### FANCA deficiency exacerbates *TgSpi1*-associated haematologic disorder

**Fig. 1:**
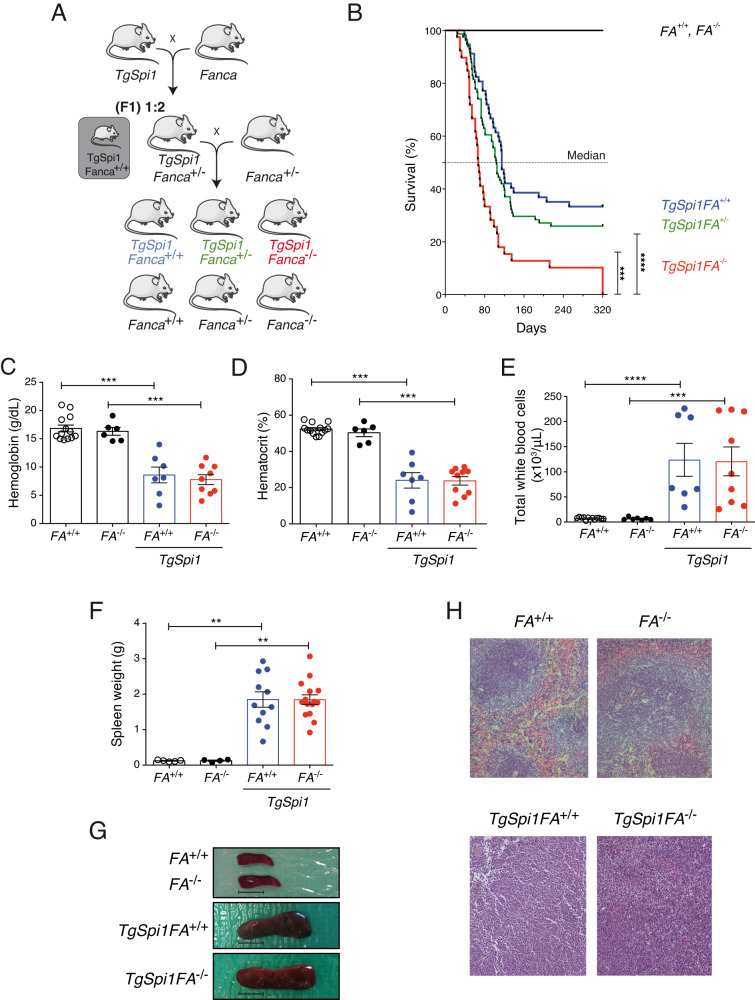
Combined genetic inactivation of *Fanca* and high expression of SPI1 accelerates anaemia and splenomegaly appearance in mice. **A** To generate mice harbouring the *Fanca* deletion and *Spi1* transgene, homozygous *Spi1* transgenic male mice (*TgSpi1*) were crossed with *Fanca*-deleted heterozygous females. From this first cross (F1), *TgSpi1* and *Fanca*-deleted heterozygous males (*TgSpi1Fanca*^*+/−*^*)* were then used for breeding with *Fanca*-deleted heterozygous females (*Fanca*^*+/−*^), and the resulting *TgSpi1* and *Fanca*-deleted homozygous (*TgSpi1FA*^−/−^) mice were identified by genotyping. Mice in grey boxes were not used. *Fanca*-deleted mice are further indicated as *FA*^−/−^ or *FA*^*+/−*^. **B** Kaplan‒Meier curve of the survival of mice bearing *TgSpi1FA*^*−/−*^ (N = 39), *TgSpi1FA*^*+/−*^ (*N* = 81) or allelic controls (*FA*^*+/+*^, *N* = 12; *FA*^*−/−*^, *N* = 12; *TgSpi1FA*^*+/+*^, *N* = 57). ****P < 0.0001, Mantel‒Cox test. Haemoglobin concentration (**C**), haematocrit level (**D**) and cell count (**E**) of peripheral blood from moribund *TgSpi1FA*^*−/−*^ and *TgSpi1FA*^*+/+*^ mice and from 3- to 4-month-old *FA*^*+/+*^ and *FA*^*−/−*^ mice. Histograms indicate the mean ± SEM from 6 to 13 individual mice according to the groups. Statistical analysis was carried out using the Mann‒Whitney test. **F** Histograms indicate the mean ± SEM of spleen weight of moribund *TgSpi1FA*^*−/−*^ and *TgSpi1FA*^*+/+*^ mice and from 3- to 4-month-old *FA*^*+/+*^ and *FA*^*−/−*^ mice from 4 to 15 individual mice according to the groups. Statistical analysis was carried out using the Mann‒Whitney test. **G** Representative images of the spleens of the mice with the 4 genotypes. Scale bar, 1 cm. **H** Haematoxylin and eosin-stained splenic sections of moribund *TgSpi1FA*^*−/−*^ and *TgSpi1FA*^*+/+*^ mice or 3- to 4-month-old healthy *FA*^*+/+*^ and *FA*^*−/−*^ mice. Magnification, ×200.

We crossed *Fanca*^+/−^ (*FA*^+/−^) mice [28] with transgenic mice constitutively expressing a *Spi1* transgene (*TgSpi1Fanca*^*+/+*^) [14] and backcrossed the first generation (F1) of *TgSpi1FA*^+/−^ mice with *Fanca*^+/−^ mice to obtain *TgSpi1FA*^−/−^ mice (Fig. 1A). The lack of *Fanca* expression was verified by RT-qPCR, and the overexpression of SPI1 was confirmed by immunohistochemistry (Supplementary Fig. S1A, B).

All mice appeared healthy at birth without congenital malformations or growth problems. At later ages, *TgSpi1FA*^*+/+*^, *TgSpi1FA*^*+/−*^ and *TgSpi1FA*^*−/−*^ mice developed anaemia and splenomegaly, leading to their death. However, all of the *TgSpi1FA*^−/−^ mice died within 10 months after birth, while only 67% of the *TgSpi1FA*^+/+^ mice died within this time frame (Fig. 1B and Supplementary Fig. S1C). The median survival of 67 days for the *TgSpi1FA*^−/−^ mice and 115 days for the *TgSpi1FA*^+/+^ mice demonstrated acceleration of disease onset (Fig. 1B). *TgSpi1FA*^*+/−*^ mice present with intermediate behaviour, with a median survival of 105 days, and 20% of the animals were still alive at 10 months of age. None of the wild-type *Fanca*^+/+^ (WT, FA^+/+^) or *Fanca*^−/−^ (FA^−/−^) mice developed a disease, indicating that the pathology requires the unrestrained expression of SPI1. At death, *TgSpi1FA*^*+/+*^ and *TgSpi1FA*^*−/−*^ mice showed a similar severity of anaemia with a reduction in both the haemoglobin and haematocrit levels (Fig. 1C, D). *TgSpi1FA*^*+/+*^ and *TgSpi1FA*^*−/−*^ mice displayed a high number of total white blood cells (>100 × 10^3^/μL) (Fig. 1E) and comparable splenomegaly (Fig. 1F, G) with disrupted splenic architecture (Fig. 1H). All these parameters indicate that their death occurs at a similar level of disease progression.

Taken together, our data demonstrate that FANCA loss of function accelerates the progression of the haemopathy associated with the unrestrained expression of SPI1 and allows for full disease penetrance.

### Lack of FANCA does not modify the lineage specificity of leukaemic cells associated with SPI1 overexpression

To shed light on how loss of FANCA function led to the observed acceleration and full penetrance of SPI1-associated disease, we searched for the haematopoietic populations contributing to the disease. At death, the proportions of CD4^+^CD8^-^ and CD4^-^CD8^+^ T lymphoid or CD11b^+^ myeloid cells in the bone marrow (BM) were similar in all genotypes, while B lymphoid, CD19^+^B220^+^, cells were equally reduced in *TgSpi1FA*^*+/+*^ and *TgSpi1FA*^*−/−*^ mice compared to non-Tg*Spi1*, *FA*^*+/+*^ and *FA*^*−/−*^ mice (Supplementary Fig. S2).

**Fig. 2:**
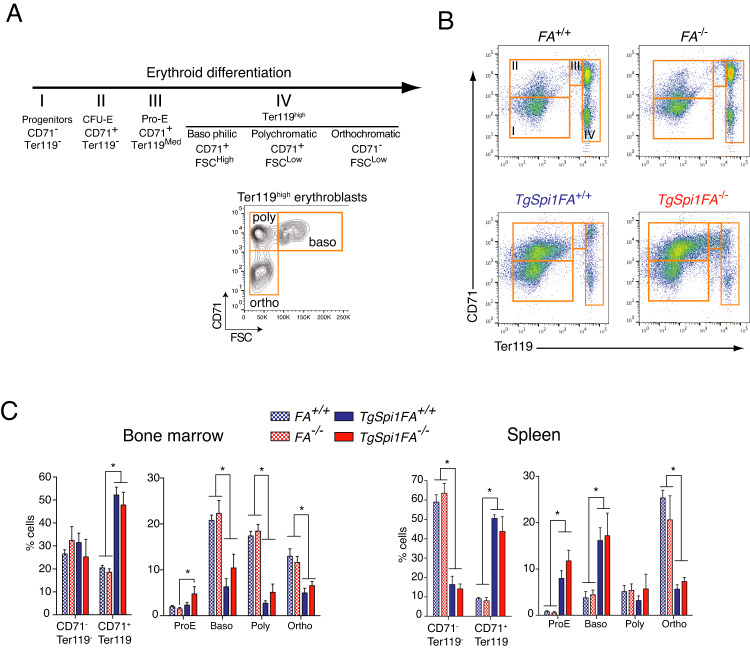
Characterization of leukaemic cells. **A** Schematic diagram representing erythroid differentiation with the specific phenotypic markers used. **B** Representative profiles of cells incubated with antibodies against Ter119 and CD71 and analysed by flow cytometry. **C** Histograms represent the mean % of each population for mice analysed (from 4 to 8 mice) according to Ter119 and CD71 markers and basophilic, polychromatic and acidophilic cells relative to 100% of bone marrow or spleen cells as described in A and B. Statistical analysis was carried out using the Mann‒Whitney test. Three types of comparisons were performed: *FA*^*−/−*^ to *TgSpi1FA*^*−/−*^; *FA*^*+/+*^ to *TgSpi1FA*^*+/+*^ and *TgSpi1FA*^*−/−*^ to *TgSpi1FA*^*+/+*^. For better visualization, only one * was written for all *P* values (*P* < 0.05), even if more highly significant.

Subsequently, we profiled erythroid subpopulations according to CD71 and Ter119 cell-surface markers and the forward scatter (FSC) parameter [34, 35]. Flow cytometry analysis identified the following six cellular subpopulations: CD71^−^Ter119^−^ (I); CD71^+^Ter119^−^ (II, colony-forming unit erythroid, CFU-E); CD71^+^Ter119^Med^ (III, pro-erythroblasts, Pro-E); and the three Ter119^High^ subpopulations (IV) that are CD71^+^FSC^High^ or basophilic, CD71^**+**^FSC^Low^ or polychromatic, and CD71^-^FSC^Low^ or orthochromatic cells (Fig. 2A, B). Compared to FA^+/+^ and FA^−/−^ mice, which show similar profiles, *TgSpi1FA*^*+/+*^ and *TgSpi1FA*^*−/−*^ mice displayed an increased proportion of CFU-E progenitors in their BM to more than 50% of the total cell count. This is at the expense of basophilic, polychromatic and orthochromatic erythroblasts, indicating erythroid differentiation blockage consistent with the observed anaemia (Fig. 2B, C). Altered erythroid differentiation was also evident in the spleen, with a clear accumulation of CFU-E progenitors in *TgSpi1* mice (Fig. 2C). Similar populations were found between *TgSpi1FA*^*+/+*^ and *TgSpi1FA*^*−/−*^ mice.

In conclusion, the proliferating blasts had an erythroid lineage origin in both *TgSpi1FA*^*+/+*^ and *TgSpi1FA*^*−/−*^ mice. Moreover, accelerated death and full penetrance of the *TgSpi1*-associated disease due to FANCA loss of function were not caused by additional alterations of other lineages such as myeloid, B or T lymphoid cells.

### FANCA deficiency promotes tumorigenicity associated with EPO independence

**Fig. 3:**
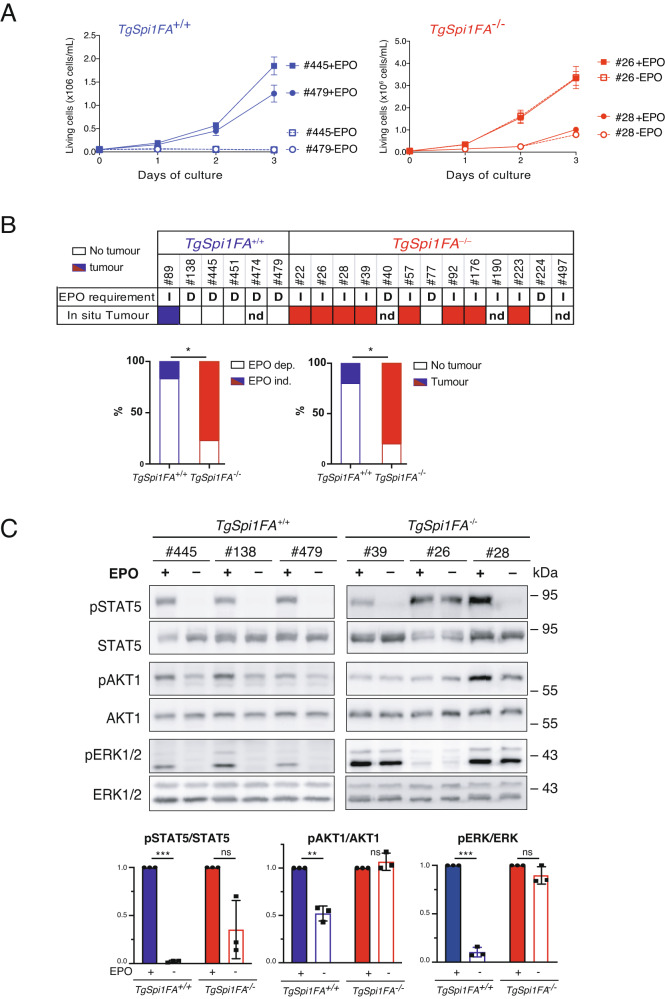
*Fanca* deletion confers EPO independence and tumorigenicity. **A** Primary cells isolated from the spleens of sick *TgSpi1FA*^*+/+*^ and *TgSpi1FA*^*−/−*^ mice with splenomegaly were cultured with (+EPO, full line) or without EPO (−EPO, dashed line). Living cells were counted on the indicated days using the trypan blue exclusion test. Data are the mean ± SEM of a minimum of 3 independent experiments. **B** EPO requirement and in situ subcutaneous secondary tumour in nude mice from *TgSpi1FA*^*+/+*^ and *TgSpi1FA*^*−/−*^ blasts. D: EPO dependent; I: EPO independent; nd: not determined. Filled boxes indicate that primary transforming cells formed secondary tumours in nude mice; empty boxes indicate that no secondary tumours were detected. Histograms represent the percentage of sick mice whose spleen was invaded by EPO dependent or EPO-independent cells among *TgSpi1FA*^*+/+*^ or *TgSpi1FA*^*−/−*^ mice. The same is shown for the ability to develop subcutaneous in situ secondary tumours. Statistical analysis was carried out using Chi-square test. * indicates *P* < 0.05. **C** Representative immunoblots of whole-cell extracts of *TgSpi1FA*^*+/+*^ and *TgSpi1FA*^*−/−*^ cells coupled with anti-pSTAT5, total STAT5, pAKT, total AKT, pERK1/2 and total ERK1/2 antibodies. Cells were cultured in the presence (+) or absence (−) of EPO for 4 h. Histograms represent the mean of the signal ratio of phosphorylated to total protein in cells deprived of EPO relative to their respective control, cells cultured in the presence of EPO from 3 independent mice. Statistical analysis was carried out using unpaired Student *t*-test. * indicates *P* < 0.05; **P < 0.01; ***P < 0,001.

It was previously shown that cells isolated from the bone marrow and spleen of sick *TgSpi1* mice required the erythroid growth factor erythropoietin (EPO) to be maintained in culture and did not give rise to tumour after subcutaneous engraftment [14]. Accordingly, EPO starvation induced growth arrest and death of blasts derived from 83% of *TgSpi1FA*^*+/+*^ mice generated in this study (Fig. 3A, left panel and 3B). In contrast, the blasts derived from only 23% of *TgSpi1FA*^*−/−*^ mice required EPO for survival and proliferation (Fig. 3A, right panel and 3B). In addition, EPO-independent cells formed in situ tumours within three weeks of their subcutaneous injection into immunodeficient mice demonstrating their ability for autonomous proliferation (Fig. 3B and Supplementary Fig. S3A). None of the EPO-dependent cells gave rise to tumours. Additionally, blasts were intravenously injected into immunodeficient mice where they were exposed to endogenous EPO. Accordingly, EPO-dependent and EPO-independent cells were able to invade the bone marrow and spleen. However, EPO-independent blasts from the *TgSpi1FA*^−*/* −^ mice displayed a strong proliferation advantage as deduced from the higher fraction of blasts in the bone marrow and spleen compared to EPO-dependent cells (Supplementary Fig. S3B). EPO-dependent cells that do not give rise to subcutaneous tumours, with no autonomous proliferation, are considered as preleukaemic cells.

To sustain blast survival and proliferation, EPO initiates several signalling pathways *via* the activation of diverse effectors, including STAT5, AKT1 and ERK1/2 [36]. Accordingly, *TgSpi1FA*^*+/+*^ preleukaemic cells cultured in the presence of EPO presented STAT5, AKT1 and ERK1/2 phosphorylation, an indicator of their activation, which was systematically reduced following EPO starvation (Fig. 3C, #445, #138, #479). In contrast, in *TgSpi1FA*^*−/−*^ blasts whose proliferation was independent of EPO, the activation of the AKT1 and ERK1/2 signalling pathways was independent of the presence of EPO (Fig. 3C, #39, #26, #28). Interestingly, the consequence of EPO deprivation on STAT5 activation differs according to the *TgSpi1FA*^*−/−*^ mice. Cells from #26 mice still presented STAT5 phosphorylation in the absence of EPO, while those from #28 and #39 mice were not able to maintain STAT5 phosphorylation, indicating that the absence of FANCA was not sufficient for STAT5 constitutive activation. Interestingly, shRNA-mediated *Fanca* downregulation in EPO-dependent *TgSpi1FA*^*+/+*^ cell lines (#138, #445, #479) was unable to confer EPO independence in vitro 9 days post-infection (Supplementary Fig. S3C, D).

In conclusion, our results show that the absence of FANCA protein contributes to the emergence of blasts that do not require EPO to survive and/or proliferate. However, the sole absence of FANCA protein in a clone is not sufficient to confer EPO independence in a short time, revealing a complex mechanism likely associated with the presence of additional epi- or genetic events.

### FANCA loss of function favours the emergence of leukaemic *TgSpi1* clones with oncogenic mutations

To gain insight into the causes of EPO independence and tumorigenicity associated with loss of FANCA function, we performed whole-exome sequencing (WES) analysis of blasts isolated from the spleen of 5 *TgSpi1FA*^*+/+*^ and 9 *TgSpi1FA*^*−/−*^ mice with anaemia and splenomegaly.

**Fig. 4:**
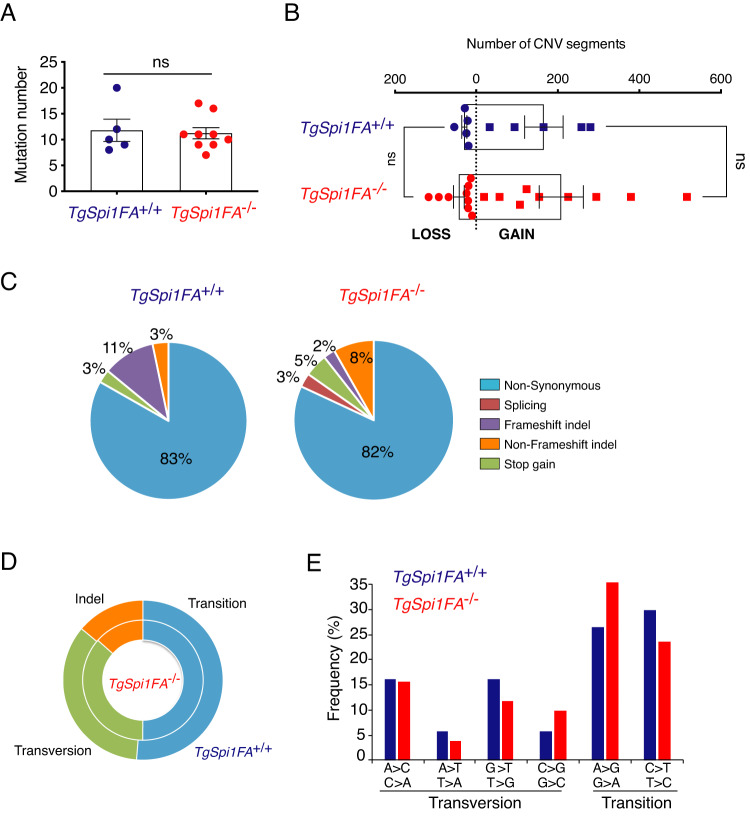
Structural genomic variations in preleukaemic and leukaemic cells. **A** Somatic mutation rates per animal (dot) are shown. Histograms indicate the mean ± SEM; ns: not significant Mann‒Whitney test. **B** Bar charts with error bars overlaid with dot plots showing the mean ± SEM of the number of CNV segments. Copy number gains and losses (>200 bp) were defined in *TgSpi1FA*^*+/+*^ preleukaemic and *TgSpi1FA*^*−/−*^ leukaemic cells using the Control-FREEC tool. Each dot represents cells from one sick mouse. Histograms indicate the mean ± SEM; ns: nonsignificant Mann‒Whitney test. **C** Pie charts showing the distribution of the type of mutations in *TgSpi1FA*^*+/+*^ (*n* = 5) or *TgSpi1FA*^*−/−*^ (*n* = 10) cells derived from sick mice. **D** Relative proportion of single nucleotide variations, transitions and transversions, and indels identified in *TgSpi1FA*^*+/+*^ (n = 5, outer circle) or *TgSpi1FA*^*−/−*^ (*n* = 10, inner circle) cells derived from sick mice. **E** Types of transitions and transversions in *TgSpi1FA*^***+****/+*^ (*n* = 5) or *TgSpi1FA*^*−/−*^ (*n* = 10) cells for all sick mice from the indicated genotypes.

WES analysis demonstrated that the number of small mutations (<200 bp) with a variant allele frequency (VAF) above 15% (Supplementary Table S1) and copy number variation (CNV) (>200 bp) (Supplementary Table S2, S3) per spleen was comparable between *TgSpi1FA*^*+/+*^ and *TgSpi1FA*^*−/−*^ mice (Fig. 4A, B). We identified a mean of 11.8 nonsilent mutations (range 8–20) for *TgSpi1FA*^*+/+*^ and 11.2 (range 7–17) for *TgSpi1FA*^*−/−*^. Qualitatively, the type (transversion vs. transition) as well as the single nucleotide variant (SNV) and indels resulting in protein variations (missense, nonsynonymous, splice site, stop-gain, frameshift and nonframeshift indels) were similar between *TgSpi1FA*^*+/+*^ and *TgSpi1FA*^*−/−*^ cells (Fig. 4C–E). Hence, even though *TgSpi1FA*^−/−^ leukaemic cells displayed higher levels of 53BP1 foci, DNA damage, chromosome breakage and intracellular reactive oxygen species (ROS) than *TgSpi1FA*^*+/+*^ preleukaemic cells (Supplementary Fig. S4), which are expected due to the lack of a functional FANCA protein, they did not show an increased level of genetic instability.

**Fig. 5:**
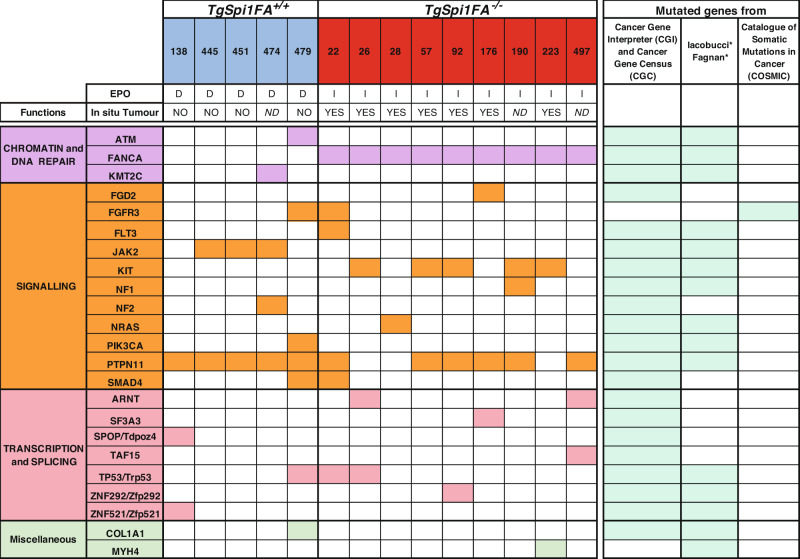
Mutated genes identified by WES in blasts of *TgSpi1FA*^*+/+*^ and *TgSpi1FA*^*−/−*^ mice. The names of genes with pathogenic mutations are shown. D: EPO-dependent clones; I: EPO-independent clones; nd no data. The ability of cells to give rise to subcutaneous tumour is indicated.

We compared the list of the 128 genes with identified variants (Supplementary Table S1) to a comprehensive list of 1081 “cancer genes” obtained by combining the Catalogue of Validated Oncogenic Mutations of the Cancer Genome Interpreter (CGI) database and the Cancer Gene Census (CGC) of the COSMIC database. Twenty-three genes displaying pathogenic mutations were identified as human cancer genes (Fig. 5 and Supplementary Fig. S5). Figure 5 also includes a variant of *Fgd2*, which is not considered to be a cancer gene in the CGI and CGC databases but has been repeatedly found to display this specific variant (at least 5 times) in the cancer samples recorded in the COSMIC database. Mutations in fifteen out of the 23 genes were also identified in human erythroleukaemia (Fig. 5) [16, 37].

Mutations in the *Fgfr3, Ptpn11, Smad4* and *Trp53* genes were identified in both genotypes that excludes that observed variants were directly responsible for the EPO independency and tumorigenicity of the *TgSpi1FA*^*−/−*^ blasts.

The JAK2 V617F mutation, which is associated with myeloproliferative disorders and EPO hypersensitivity [38], was identified exclusively in blasts of *TgSpi1FA*^*+/+*^ mice, including #445 and #451, with VAF values of 28 and 18%, respectively. JAK2 Y931C is another mutation conferring gain of function [39] and was found in #474 mice. However, none of the JAK2 mutations conferred per se a proliferative advantage allowing in vivo clonal selection.

Interestingly, mutations in *Flt3, Fgd2*, *Nras and Kit* were identified exclusively in *TgSpi1FA*^*−/−*^ cells. All of these genes are key oncogenes in leukaemia. The *Kit* D818Y (#26, #57, #92, #223) or *Nras* Q61H (#28, #39) mutations, identified in 45 and 20% of mice, respectively, have been previously shown to confer growth factor independency in the *TgSpi1FA*^*+/+*^ cells or myeloma cells, respectively [15, 40], indicating a causal relationship between constitutively activated KIT and RAS pathways and growth factor independence.

Our results show that the somatic mutation rate was similar between *TgSpi1FA*^*+/+*^ and *TgSpi1FA*^*−/−*^ mice, but specific mutations in known oncogenes activating signalling pathways, *Kit* and *Ras*, were identified in FANCA-deficient cells. Considering the stochasticity of the mutational process, these data suggest that FANCA deficiency supports the selection of oncogenic mutations known to favour cell overgrowth. This hypothesis predicts that the oncogenic mutations identified in *TgSpi1FA*^*−/−*^ leukaemic cells should be present in the cell populations issued from the bone marrow or spleen of the *TgSpi1FA*^*+/+*^ mice in low-prevalence subclones. Accordingly, searching for *Kit or NRas* variants with low VAF in the preleukaemic cells from TgSpi1FA^+/+^ mice, we found KIT D818Y (mice #445 VAF 1.9%*)* and NRAS Q61H or G60V mutations (mice #479 VAF 2.1% and mice #138 VAF 1.6%, respectively). However, such mutated cells did not expand to become a major population of the spleen of *TgSpi1FA*^*+/+*^ mice displaying active FANCA, even when maintained in culture for several months.

Altogether, our results indicate that loss of FANCA establishes a favourable cellular environment for the selection of cells carrying genes with oncogenic mutations.

### A unique transcriptional deregulation is associated with the leukaemic status of FANCA-deficient cells

**Fig. 6:**
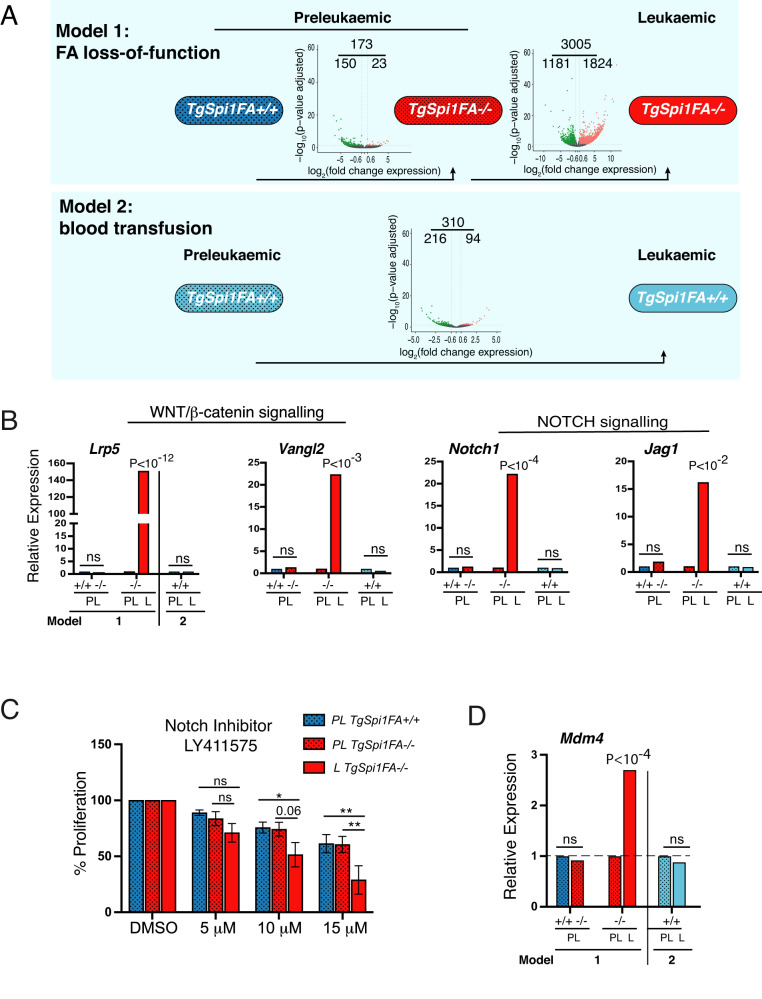
Loss of FANCA strongly impacts the transcriptome of leukaemic cells. **A** Top, model 1 (FA loss-of-function): volcano plots of differentially expressed genes between the preleukaemic *TgSpi1FA*^*+/+*^ versus preleukaemic *TgSpi1FA*^*−/−*^ cells or between the *TgSpi1FA*^*−/−*^ preleukaemic and leukaemic cells. Bottom, model 2 (blood transfusion): volcano plots of differentially expressed genes between the *TgSpi1FA*^*+/+*^ preleukaemic versus leukaemic cells. Increased genes in leukaemic cells (FC ≥ 1.5, *P* < 0.05) are indicated in red; repressed genes (FC ≤ 0.67, *P* < 0.05) are indicated in green. **B** Relative expression of genes from NOTCH or WNT/β-catenin pathways measured by RNA-seq between leukaemic (L) and preleukaemic (PL) cells from mice with the indicated genotypes. *TgSpi1FA*^*+/+*^(#138, #445, #451, #479) or *TgSpi1FA*^*−/−*^ (#40, #77, #224) preleukaemic cells or *TgSpi1FA*^*−/−*^ leukaemic cells (#26, #28, #39). Adjusted P values for multiple testing using the Benjamini and Hochberg method are indicated. ns: not significant. **C** *TgSpi1FA*^*+/+*^(#138, #445, #451, #479) or *TgSpi1FA*^*−/−*^ (#40, #77, #224) preleukaemic cells or *TgSpi1FA*^*−/−*^ leukaemic cells (#26, #28, #39) were seeded at 2 × 10^4^ cells/mL and treated with the indicated doses of the NOTCH inhibitor (LY411575). Living cells were counted 3 days post-treatment using a DAPI exclusion test and quantified as a percentage of the control DMSO. Data are the mean ± SEM of 3 independent experiments. Statistical analysis was carried out using two-way ANOVA against the DMSO control sample. * indicates *P* < 0.05; **P < 0.01. PL preleukaemic, L Leukaemic. **D** Relative expression of *Mdm4* mRNA measured by RNA-seq in preleukaemic or leukaemic cells of mice with the indicated genotypes (PL preleukaemic, L leukaemic). *P* values (RNA-seq analysis) were adjusted for multiple testing using the Benjamini and Hochberg method.

We hypothesized that transcriptomic modifications may contribute to the selection of leukaemic mutated clones in the absence of FANCA. We first compared RNA-seq data between *TgSpi1FA*^*−/−*^ leukaemic and preleukaemic cells (model 1). To exclude interference due to EPO signalling, we performed RNA-seq on blasts cultured in the presence of EPO. We identified 3005 differentially regulated transcripts (|FC| ≥ 1.5; *P*_adjusted_ < 0.05), of which 1181 were repressed and 1824 were increased, in *TgSpi1FA*^*−/−*^ leukaemic compared to *TgSpi1FA*^*−/−*^ preleukaemic cells (Fig. 6A, model 1 and Supplementary Table S4).

GSEA analysis revealed that leukaemic cells were significantly enriched for functional annotations related to WNT/β-catenin signalling that also include genes of NOTCH signalling (Supplementary Fig. S6A). Relative expression of *Lrp5, Vangl2, Notch1* or *Jag1* analysed by RNA-seq experiments are shown as examples of DEG encoding proteins of the WNT/β-catenin or NOTCH signalling, respectively (Fig. 6B, middle red bars), and was validated by RT-qPCR in the *TgSpi1FA*^*−/−*^ leukaemic compared to *TgSpi1FA*^*−/−*^ preleukaemic cells (Supplementary Fig. S6B). Interestingly, only 173 transcripts were differentially expressed between the *TgSpi1FA*^*+/+*^ and *TgSpi1FA*^*−/−*^ preleukaemic cells (Fig. 6A), and none of the genes of the WNT/β-catenin and NOTCH signalling pathways were deregulated (Fig. 6B and Supplementary Table S5), indicating a specific modification of the transcriptional profile in the FANCA-deficient leukaemic cells.

We next sought to determine whether the transcriptional deregulation was specific to the FANCA-depleted context. One strategy to answer this question is to characterize genes differentially expressed between preleukaemic and leukaemic *TgSpi1FA*^*+/+*^ cells. However, the rarity of leukaemic *TgSpi1FA*^*+/+*^ mice in model 1 (Fig. 3B) prevents such analysis. To resolve this question, we used another previously generated model of leukaemic progression initiated from *TgSpi1FA*^*+/+*^ preleukaemic mice (leukaemic model 2) [14, 15], whose leukaemic cells emerged after serial blood transfusions of preleukaemic *TgSpi1FA*^*+/+*^ mice. Blood transfusion lowered circulating EPO levels in anaemic mice and triggered in vivo selection and expansion of EPO-independent and tumorigenic cells with KIT gain-of-function mutations [14, 15]. Therefore, in this model, blood transfusion allows the emergence of leukaemic cells, instead of FANCA loss of function.

Only 310 transcripts were differentially expressed between preleukaemic and leukaemic *TgSpi1FA*^*+/+*^ cells (Fig. 6A, model 2, Supplementary Table S6). Among them, 4 increased genes and 29 repressed genes were also found to be differentially expressed between preleukaemic and leukaemic *TgSpi1FA*^*−/−*^ cells (model 1), none of which belonged to the WNT/β-catenin or NOTCH signalling pathways (Supplementary Table S7, Fig. 6B and Supplementary Fig. S6B, model 2). Therefore, FANCA deficiency is associated with a specific transcriptomic programme in leukaemic cells. Consistent with the specific transcriptional modification, the *TgSpi1FA*^*−/−*^ leukaemic cells displayed hypersensitivity to inhibition of NOTCH signalling compared to preleukaemic *TgSpi1FA*^*−/−*^ or *TgSpi1FA*^*+/+*^ cells (Fig. 6C). These data demonstrate the requirement of NOTCH signalling in the long-term proliferation of *TgSpi1FA*^*-/ -*^ leukaemic cells.

Recent demonstration shows that *MDM4* amplification in the bone marrow cells of FA patients confers greater fitness to the cells and yields clonal expansion preceding transformation to myeloid leukaemia [41]. In agreement, we found a higher expression of *Mdm4* mRNA in the *TgSpi1FA*^*−/−*^ leukaemic compared to preleukaemic cells, that was not observed in the leukaemic *TgSpi1FA*^*+/+*^ mice (Fig. 6D, model 1 *versus* model 2). These data reinforce the hypothesis that FANCA deficiency leads to transcriptional changes that are required for leukaemic transformation and suggest that, as observed in FA patients, MDM4 contributes to the proliferative advantage of FA murine leukemic cells [41].

**Fig. 7:**
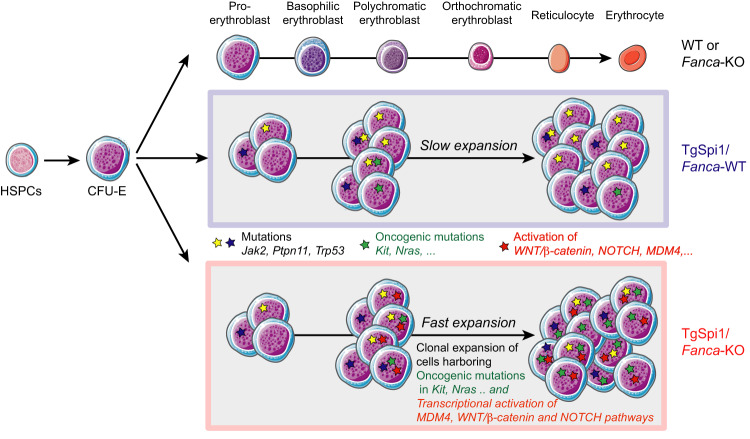
Schematic representation of the consequences of FANCA loss of function in leukaemic progression of *TgSpi1* erythroproliferative syndrome. WT and *Fanca*-deficient mice exhibit normal erythropoiesis (top). SPI1 overexpression blocks erythroid differentiation at the CFU-E stage and maintains the proliferation of progenitors, allowing the accumulation of spontaneously arising mutations without leading to growth factor independence (middle). The additional absence of the DDR protein FANCA increases the transcription of genes encoding proteins favouring proliferation pathways, such as MDM4, NOTCH and WNT/*β-*catenin and favours the expansion of clones harbouring *Kit* or *Nras* oncogenic mutations conferring EPO independency. This combination of events underlies a rapid leukaemic progression (bottom).

In conclusion, we demonstrate a peculiar transcriptional deregulation associated with the leukaemic status of cells devoid of FANCA activity. We propose that these specific transcriptional modifications represent a key point participating in the clonal selection of *TgSpi1FA*^*−/−*^ leukaemic cells by conferring advantage to the cells with secondary mutations in *Kit* or *Ras* genes (Fig. 7).

## Discussion

This work provides unexpected evidence that deficiency in the FANCA DDR protein fosters leukaemic progression not by increasing the rate of mutations but by allowing the proliferation of cells with oncogenic variants associated with a reprogrammed transcriptomic landscape.

Although preleukaemic erythroblastic cells expressing high levels of SPI1 exhibit replicative stress [32, 33], the additional deficiency in DNA repair and replication rescue by FANCA loss-of-function did not further increase the gene and genomic instability of the cells. This outcome is in agreement with published data demonstrating that FANC pathway deficiency leads to a reduced mutational load in surviving cells both in vitro and in vivo [42–45]. A recent study performing exome sequencing on FA patient-derived hematopoietic cells with clonal evolution also showed that FA patient samples did not display more somatic mutations than control AMLs [41].

Leukaemic *TgSpi1* cells due to FANCA loss-of-function displayed constitutive activation of EPO signalling pathways that supported their proliferation, similarly to the blood transfusion-derived leukaemic *TgSpi1* cells with a normal FANC pathway [14]. In both models, EPO independency and tumorigenicity were associated with activating mutations in genes encoding key signalling proteins, i.e., *Kit or Nras*. Cells with NRAS activation displayed strong and/or constitutive activation of ERK and AKT, respectively, while STAT5 was not constitutively increased. In contrast, KIT activation was associated with constitutive activation of ERK, AKT and STAT5. These results support the major function of different oncogenic mutations in the autonomous proliferation and survival of leukaemic cells, together with FANCA depletion and SPI1 overexpression. Our work demonstrated the existence of cells with *Kit and Nras* mutations with a low VAF in EPO-dependent, preleukaemic cells isolated from *TgSpi1FA*^*+/+*^ mice. These cells rarely spontaneously invade the bone marrow or spleen of mice, in contrast to *TgSpi1FA*^*−/−*^ mice. The leukaemic progression from the *TgSpi1* model described by Moreau-Gachelin et al. [14] (leukaemic model 2) were due to serial blood transfusion in anaemic animals that transitorily palliates to their anaemic status and blocks EPO production. This established a selective pressure allowing in vivo expansion of rare EPO-independent cells with *Kit* mutations [15]. In contrast, *TgSpi1FA*^*−/−*^ mice develop spontaneously overt leukaemia. Such observations strengthen the hypothesis that leukaemic transition in *TgSpi1FA*^*−/−*^ mice is sustained by additional changes in the intracellular or extracellular environment, which favours the expansion and selection of cells with oncogenic mutations. Our findings show that in contrast to leukaemic cells with normal FANCA, cells devoid of FANCA and mutated in signalling oncogenes displayed a reprogrammed transcriptional landscape. We hypothesized that transcriptional reprogramming might be involved in the emergence of leukaemic cells. Examples of the contribution of transcriptional deregulation to cellular expansion in oncogene mutated cells are reported in the literature. Indeed, transcriptional deregulation may favour the expansion of cells with NRASG12D oncoproteins whose proliferation required a reduction in *Spry2* transcription, encoding a negative regulator of the RAS signalling pathway and, thus represents an additional event, which augments MAPK signalling and contributes to transformation [46]. Similarly, the finding that epiallele, referring to variation in methylation of CpGs in discrete sets and differing among AML cells, may have functional consequences on transcription is of significance [47]. Indeed, it was recently proposed that this epiallele and, thus, transcriptional diversity can occur prior to overt transformation, possibly enabling preleukaemic cells to overt leukaemogenesis [48].

It was recently reported in FA patients with clonal evolution of bone marrow cells that 1q trisomy is the most frequent chromosomal event, driving enforced MDM4 oncogene expression through gene dosage and attenuating the p53 anti-cancer barrier in FA [41]. Such p53 attenuation confers a keen advantage to MDM4 overexpressing cells in FA cells, triggering stem/progenitor cell survival and clonal expansion but is not transforming per se; additional oncogenic mutations are required (3q+, *RUNX1, RAS…*). In the erythroleukaemic model used in our study, we also found overexpression of *Mdm4* during the emergence of FANCA-depleted leukaemic clones, as well as additive mutations, including *Ras* and *Kit* genes. Additionally, FANCA loss of function in leukaemic SPI1-overexpressing cells deregulates a complex network of genes known for their strong association with leukaemia development. In particular, several membrane receptors/ligands or downstream signalling pathways, including WNT/β-catenin and NOTCH signalling, were transcriptionally deregulated in *Fanca-*deleted leukaemic cells. Increased expression of WNT/β-catenin or NOTCH and associated pathways has already been reported in FANCC-deficient B lymphoid human and mouse cells [49, 50]. Nevertheless, *Fanca* deletion alone in *TgSpi1* preleukaemic cells or the presence of oncoproteins of signalling pathways alone in *TgSpi1* leukaemic cells with functional FANCA were not sufficient to strongly activate WNT/β-catenin or NOTCH pathways. These data reveal a strict association between transcriptomic deregulation in cells with FANCA loss-of-function and activating mutations of signalling pathways. We suggest that MDM4, WNT/β-catenin or NOTCH signalling participates in the selection and expansion of cells with constitutively increased KIT or RAS signalling pathways.

Accordingly, activation of the β-Catenin pathway was recently demonstrated to be responsible for the preleukaemic to AML transition in distinct preleukaemic diseases due to MDMX overexpression [13]. In the *TgSpi1FA*^*−/−*^-derived leukaemia cells, the inhibition of WNT/β-catenin signalling did not affect cell proliferation, at least in vitro, consistent with a role of WNT/β-catenin signalling in the preleukaemic to leukaemic transition rather than in leukaemia maintenance. In contrast, the NOTCH pathway was required to maintain the proliferative status of *TgSpi1FA*^*−/−*^ leukaemic cells. Future studies are required to characterize the mechanisms responsible for the overactivation of genes controlling proliferation pathways. One hypothesis to investigate is WNT/β-catenin and NOTCH signalling, such as MDM4, plays a role in repression of the p53 anti-tumoral barrier in FA^−/−^ cells.

Together with reported studies [41, 51], our work supports that transcriptional deregulation is one of the mechanisms by which the absence of Fanconi proteins may contribute to abnormal haematopoietic cell function. We propose that cells with FANC deficiency are prone to transcriptional modifications of genes encoding proteins participating in multiple signalling pathways, favouring the expansion of clones carrying *Ras* or *Kit* oncogenic drivers and thus supporting leukaemogenesis (Fig. 7).

Although it remains to be determined how these functions ultimately exert a positive effect on the emergence of cells carrying oncogenic mutations, they represent targets that can be disrupted to subvert leukaemic progression.

## Methods

### Mice and cell culture

*Fanca*^*+/−*^ and *TgSpi1* mice were derived from the FVB/N and DBA2/J backgrounds, respectively [14, 28]. Mice deficient in the *Fanca* gene and expressing *TgSpi1* (*TgSpi1Fanca*^−/−^ or *TgSpi1FA*^−/−^) were generated by mating *Fanca*^+/−^ mice with homozygous *TgSpi1* mice to obtain double-heterozygous mice (F1) (Fig. 1A). These F1 mice were then mated with *Fanca*^+/−^ mice to generate mice harbouring homozygous mutations of *Fanca* and heterozygous *TgSpi1*. The project was officially approved by the Animal Experimentation Ethics Committee (registered by the French Department of Research) and conducted in accordance with French laws and regulations. The resulting mice of the indicated genotypes were monitored for survival. When needed, moribund mice were sacrificed for analysis of different biological parameters.

Preleukaemic and leukaemic cells derived from *TgSpi1* mice were obtained previously [14, 15].

For NOTCH inhibition, cells were treated for 3 days with LY411575 (Sigma‒Aldrich), a NOTCH and γ secretase inhibitor. The number of viable cells was determined with DAPI labelling (0.5 µM) in 96-well plates by the BD High-Throughput Sampler (HTS) option from the CytoFLEX flow cytometer (Beckman Coulter). Flow cytometry data were analysed using FlowJo v10.6.2 software (Tree Star).

### Real-time quantitative PCR (RT-qPCR)

Total RNA was isolated from cells using the RNeasy Plus Mini Kit or Maxwell RSC simplyRNA Cell Kit (Promega), and RT‒qPCR was performed using TaqMan or SYBR Green technology as described in detail in the Supplementary data.

### Bone marrow and spleen immunophenotyping by flow cytometry

Single-cell suspensions of freshly collected bone marrow cells were immunostained with APC-H7 (560185, BD Biosciences) or APCeFluor780 (eBioscience 47-1172-82) -conjugated rat anti-KIT, FITC-conjugated rat anti-CD71 (553266, BD Biosciences), APC-conjugated rat anti-Ter119 (557909, BD Biosciences) and PE-conjugated streptavidin (554061, BD Biosciences) and biotin-conjugated rat anti-CD123 (555070, BD Biosciences) antibodies for the detection of erythroid progenitor (CFU-E) cells, as previously described [52]; FITC-conjugated rat anti-CD11b (553310, BD Biosciences), PerCP-Cy^TM^5.5-conjugated rat anti-mouse CD19 (45-0193-82, eBioscience) and PE-conjugated rat anti-mouse CD4 (553049, BD Biosciences), and APC-conjugated rat anti-myeloid CD8 (553035, BD Biosciences) and PE-Cy^TM^7- conjugated rat anti-myeloid B220 (25-0452-82, eBioscience) antibodies were used for the detection of freshly collected bone marrow cells. Stained cells were analysed with a BD Accuri C6 or a Fortessa analyser (BD Biosciences), and FlowJo (Tree Star) was used for data acquisition and analysis.

Additional details of the methods are included in the supplementary data.

## Supplementary information

Supplementary File

Supplementary Table S1

Supplementary Table S2

Supplementary Table S3

Supplementary Table S4

Supplementary Table S5

Supplementary Table S6

Supplementary Table S7

## Data Availability

The high-throughput data generated are provided in the Gene Expression Omnibus under accession number GSE164411.
