# Supplementary material for: FANCA deficiency promotes leukaemic progression by allowing the emergence of cells carrying oncogenic driver mutations
Source: Oncogene. 2023 Aug 12;42(37):2764–75. doi: 10.1038/s41388-023-02800-9 (PMC10491493; doi:10.1038/s41388-023-02800-9)
Supplement: Supplementary file 1 — Supplementary File [file 41388_2023_2800_MOESM1_ESM.docx]

**SUPPLEMENTAL DATA**

**FANCA deficiency promotes leukaemic progression by allowing the emergence of cells carrying oncogenic driver mutations**

Patrycja Pawlikowska, Laure Delestré, Sebastian Gregoricchio, Alessia Oppezzo, Esposito Michela, M’Boyba Diop, Filippo Rosselli and Christel Guillouf

**SUPPLEMENTARY METHODS**

***Real-time quantitative PCR (RT‒qPCR)***

To evaluate *Fanca* expression in mice or after *Fanca* shRNA transduction in cells, RT‒qPCR was performed using TaqMan. RNA (150 ng) was used to prepare cDNA using Superscript IV Reverse Transcriptase (Life Technologies). qPCR was performed on 5 ng of cDNA in duplicate on the 7500 RT‒qPCR System (Applied Biosystems) using TaqMan Gene Expression Assays (Applied Biosystems) (*Polr2α*, Mm00839493_m1; *Gapdh*, Mm99999915_g1; *FancA*, Mm01243365_m1).

To validate the RNA-seq results, RT‒qPCR was performed using SYBR Green method. RNA (500 ng) was reverse-transcribed using a RevertAid First Strand cDNA Synthesis Kit (Thermo Scientific), and qPCR was performed using iTaq Universal SYBR Green Supermix (Bio-Rad) on 6.25 ng of cDNA.

For the two methods, the mRNA levels were normalized to the *Polr2α* and/or *Gapdh* mRNA levels (ΔCt=Ct*Gene*-Ct*Polr2a* or Ct*Gapdh*). The relative mRNA expression was calculated relative to the indicated control by the comparative Ct method using the formula 2^−ΔΔCt^. When expression analysis was compared between mice from different genotypes and, therefore individual mice from the same genotype represents biological replicates, expression relative to the control was performed using the formula calculated from the mean of the ΔCt, reason why the SEM are indicated on the ΔCt.

***Western blotting***

Analysis of cell extracts by Western blotting was performed as described previously [1]. Primary antibodies against phospho-pAKT (Cell Signalling, 4060), AKT (Cell Signalling, 4685), phospho-STAT5 (Cell Signalling, 9351), STAT5 (Santa Cruz, SC835), phospho-p44/42 MAPK (ERK1/2) (Thr202/Tyr204) (E10) (Cell Signalling, 9106) and p44/42 MAPK (ERK1/2) (Cell Signalling, 9102) were used for immunostaining. Specific peroxidase-conjugated secondary antibodies (Jackson Laboratory) were used to detect protein expression by an LAS-4000 imager (GE Healthcare) using an enhanced chemiluminescence kit (Pierce). Images were cropped using Photoshop software (Adobe Systems France).

***Blood sample analysis***

Peripheral blood was collected from mice via their submandibular vein into EDTA-coated tubes, and cells were counted on an MS9-5 haematology counter (Melet Schloesing Laboratory).

***Immunohistochemistry***

Following fixation of the mouse femurs or spleens in 4% paraformaldehyde, paraffin sections (4 μm thick) were treated with xylene and then rehydrated. Heat-induced epitope retrieval was achieved with pH 6 citrate buffer at 98°C for 30 min. Endogenous peroxidase activity was quenched by 3% H_2_O_2_ for 10 min. The sections were placed on coverslips (Shandon) and incubated with blocking serum then with a rabbit anti-SPI1 antibody (Cell Signalling, clone 9G7, 2258) in blocking serum (1/10) for 1 hr. Detection was performed using the Rabbit PowerVision kit (ImmunoVision Technologies), and the signal was revealed with DAB peroxidase substrate solution. Cell nuclei were counterstained with Mayer’s haematoxylin solution, and finally, the sections were dehydrated and mounted (Diamount). Bone marrow or spleen sections from each animal were scanned using a virtual slide system (VS120, Olympus) with a 20X objective lens.

***Immunofluorescence***

Cells were cytospinned on poly-L-lysine–coated slides or on glass Laboratory-Tek slides (Thermo Scientific) coated with SuperFibronectin (Sigma-Aldrich) and fixed with 4% paraformaldehyde in PBS for 15 min at room temperature, permeabilized in 0.5% Triton X-100 in PBS for 10 min and blocked with 3% BSA in PBS containing 0.05% Tween-20 for 20 min. For indirect immunofluorescence experiments, incubations with the primary antibody rabbit anti-53BP1 (Abcam, ab175933) were performed overnight at 4°C, followed by incubations for 1 h with Alexa 568-conjugated secondary antibodies (A-11036, Invitrogen) at room temperature and in the dark. Slides were air-dried and mounted using Fluoromount G (Southern Biotech) with DAPI. Images were captured using an Axiocam Mrm camera (Carl Zeiss) connected to an Axio observer Z1 microscope (Carl Zeiss) with a 63x/1 oil immersion objective. Acquisitions were made using Axiovision software (Carl Zeiss). The images were then imported into ImageJ, pseudocolored and merged using the same software.

***Metaphase chromosome staining***

Erythroid blast cells were incubated with colcemid (0.1 μg ml−1) for 3 hours at 37°C, and metaphase spreads were obtained using standard procedures as previously described [2]. Briefly, cells were incubated in hypotonic solution (0.075 M KCl) for 15 min at 37°C, and the reaction was stopped by adding freshly prepared fixative solution (3:1 methanol/glacial acetic acid), followed by spreading on SuperFrost Plus slides (Thermo Scientific). Fluorescence in situ hybridization (FISH) analysis was performed according to the manufacturer’s protocol (Telomere PNA FISH Kit/Cy3, Dako). Metaphases from each sample were blindly scored for chromosome deletions, gaps, fragments and fusions.

***Cell transplantation into immunodeficient recipient mice and EPO dependency assays***

Subcutaneous transplantation was performed with 10^7^ cells from splenic cells derived from splenomegaly into Swiss nude mice as previously described [3] (three mice per assay). Tumour nodules were usually visible 3 weeks after the graft when the tumour mass reached a diameter of 0.5 cm.

Blasts derived from bone marrow or spleen of sick mice were transplanted through intraveinous injection in NOD-scid IL2Rgammanull (NSG) sublethally (1.5Gy) irradiated recipient mice and expansion of erythroblasts was analysed in the bone marrow.

For the EPO dependency test, cells were plated at 1 000 000 cells/mL either with or without EPO for at least 2 weeks. Cells able to survive and expand in the absence of EPO (1U/mL) after 2 weeks were considered to be “EPO independent”; otherwise, they were defined “EPO dependent” cells.

***Virus production***

For lentiviral production, HEK293T cells (Thermo Scientific) were plated in 100-mm poly-L-lysine-coated plates (BD Biosciences) with DMEM complete media (containing 10% FBS, 1% L-glutamine and penicillin-streptomycin) (Life Technologies). The plasmid mixture was prepared for cotransfection following the manufacturer’s protocol for the Lipofectamine 2000 transfection reagent (Life Technologies). The cotransfection mixture consisted of a plasmid containing shRNA *Fanca* (pGIPZ lentiviral shRNA *Fanca*, Thermo Fisher Scientific, V3LMM_438296, AACACAACGAACTGAAACT) with packaging helper virus plasmids. One day after transfection, the medium was changed to fresh DMEM complete medium for virus production. Forty-eight hours posttransfection, lentivirus-containing supernatants were retrieved by filtering through 0.45 μm cellulose acetate filters and concentrated using ultracentrifugation.

***Lentiviral Transduction***

*TgSpi1FA^+/+^* erythroblasts were plated at a concentration of 10^6^ cells/mL in complete media supplemented with EPO (1 U/mL) and transduced with viral particles. Eight hours later, the cells were diluted to a concentration of 10^5^ cells/mL. Forty-eight hours postinfection, GFP-positive cells were sorted using a FACSAria instrument (BD Biosciences) and expanded in complete media supplemented with EPO (1 U/mL) for future experiments.

***Primers for RT-qPCR***

The following primers (Eurogentec) were used for qPCR using SYBR green: GAPDH (forward 5'-CATCACTGCCACCCAGAAGACTG-3' and reverse 5'-ATGCCAGTGAGCTTCCCGTTCAG-3'); *Notch1* (forward 5’-GCTGCCTCTTTGATGGCTTCGA-3’ and reverse 5’- CACATTCGGCACTGTTACAGCC-3’); *Lrp5* (forward 5’-CCTCACCATTGATTATGCCGACC-3’ and reverse 5’-GATCGTCAGCTATCACCATGCG-3’); *Vangl2* (forward 5’-GCATCCTTCAGCACCTGGAGTT-3’ and reverse 5’-TCGCTCACCAAGGTCCACTGTT-3’).

***RNA-seq***

RNA-seq libraries were generated from 300 ng of total RNA with the Illumina® TruSeq® RNA Sample Preparation Kit v2 (Part Number RS-122-2001). One hundred base-pair paired-stranded reads were sequenced on an Illumina HiSeq 4000 system with multiplex sequencing. Sequencing was performed to obtain at least 2x50x10^6^ reads for each sample. Sequence reads were mapped onto the mm10/GRCm38 assembly of the mouse genome using STAR v2.5.3a [4]. Gene expression was quantified using htseq-count v0.6.1p1 [5] with gene annotations from Ensembl release 98. Statistical analysis was performed using R (3.3.2). Read counts were normalized across samples with the median-of-ratios method proposed by Anders and Huber [6] to make these counts comparable between samples. Differential gene expression analysis was performed using the method proposed by Love et al. [7] and implemented with the Bioconductor package DESeq2 version 1.16.1. P-values were adjusted for multiple testing using the Benjamini and Hochberg method.

Pathway analyses were performed using STRING v11 with the highest confidence interaction score (0.9) [8] .

Enrichment analyses were performed using gene set enrichment analysis (GSEA) v4.1 [9]. All gene sets in MSigdb v7 have been investigated from RNA-seq data [10].

### *Whole-exome sequencing*

### Genomic DNA was isolated from cells using the QiAamp DNA Micro Kit (Qiagen). Two hundred nanograms of genomic DNA was sheared with the Covaris E220 system (LGC Genomics/Kbioscience). DNA fragments were end-repaired, extended with an 'A' base on the 3’ end, ligated with paired-end adaptors with the Bravo Platform (Agilent) and amplified (ten cycles). Exome-containing adaptor-ligated libraries were hybridized for 40 h with biotinylated oligo RNA baits using SureSelect Mouse All Exon (Agilent) and enriched with streptavidin-conjugated magnetic beads. The final libraries were indexed, pooled and sequenced using the onboard cluster method as paired-end sequencing (2x100 bp reads) on an Illumina HiSeq 2000 sequencer on the Gustave Roussy genomic platform.

***Bioinformatics analysis of Whole-Exome Sequencing***

Whole-exome sequence reads were mapped to the mouse genome build mm9 by using the Burrows-Wheeler Aligner MEM algorithm (BWA version 0.7.10)[11] with the following parameter: -A 2. For compatibility with the Genome Analysis Toolkit (GATK), the read group ID was attached to every read in the resulting alignment file (bam file) with the -R parameter, and shorter split hits were marked as secondary with -M. The duplicate reads were removed by Picard MarkDuplicates (version 1.119 - “Picard Toolkit” 2019, Broad Institute, GitHub Repository (<http://broadinstitute.github.io/picard/>) using a position sorted bam file. Alignments around indels were corrected using GATK 3.2 RealignerTargetCreator followed by GATK IndelRealigner. Base quality score recalibration (BQSR) was performed with GATK BaseRecalibrator followed by GATK PrintReads. In the first pass of the BQSR method, the parameter -knownSites was used to specify one database of known polymorphic sites: dbSNP128. Samtools version 0.1.18 [12] was used to generate mpileup files from bam files obtained in previous steps using the following parameters: -A -B -q 20.

***Detection of Single Nucleotide Variant***

Somatic short variant (SNV and indel) calling was performed using Varscan somatic version 2.3.7 [13] in tumour samples with DNA from the mouse tail as a reference sample with the following parameter: --min-var-freq 0.1. Only variants with a P value <0.0001 were kept.

ANNOVAR program version 141112 was used to annotate variants [14]. In this tool, the relevant transcripts are reported based on the refGene database [15]. The presence of the variant in dbSNP128 and/or in FVBNJ and DBA2J strains from Mouse Genome Project Release 1211 was added to the annotations [16]. We excluded synonymous SNVs, polymorphisms (SNPs) as defined in dbSNP 128, variants located in intergenic, intronic, untranslated regions and noncoding RNA regions.

### Selection of *Single Nucleotide Variant*

The selection of functional variants reported in Figure 5 was performed by searching the mutated candidates among the comprehensive list of “cancer genes”, including the Catalog of Validated Oncogenic Mutations of the Cancer Genome Interpreter (CGI) database [17] and the Cancer Gene Census (CGC) of the COSMIC (v92) database [18]. If mutated genes were not found in these two categories, we selected the genes if the variants were observed at least 5 times among the curated database of somatic mutations of COSMIC [19]. All variants were confirmed through visualization with IGV (2.3.88).

### *Detection of Copy Number variation (CNV)*

CNV was identified with Control-FREEC version 9.1 (from pileup files generated using samtools v0.1.18) with the following parameters: breakPointThreshold = 0.8, breakPointType=2, degree = 1, minCNAlength = 1, noisyData = TRUE, ploidy = 2, readCountThreshold = 50 [20].

CNV details for each sample are described in Supplementary Table S3.

Supplementary Table S2 presents the gain and loss of genomic DNA in each gene. When both were found in the same gene, they were indicated as gain+loss.

**References**

1	Delestre L, Cui H, Esposito M, Quiveron C, Mylonas E, Penard-Lacronique V *et al*. Senescence is a Spi1-induced anti-proliferative mechanism in primary hematopoietic cells. *Haematologica* 2017; 102: 1850-1860.

2	Naim V, Rosselli F. The FANC pathway and BLM collaborate during mitosis to prevent micro-nucleation and chromosome abnormalities. *Nature cell biology* 2009; 11: 761-768.

3	Moreau-Gachelin F, Wendling F, Molina T, Denis N, Titeux M, Grimber G *et al*. Spi-1/PU.1 transgenic mice develop multistep erythroleukemias. *Mol Cell Biol* 1996; 16: 2453-2463.

4	Dobin A, Davis CA, Schlesinger F, Drenkow J, Zaleski C, Jha S *et al*. STAR: ultrafast universal RNA-seq aligner. *Bioinformatics (Oxford, England)* 2013; 29: 15-21.

5	Anders S, Pyl PT, Huber W. HTSeq--a Python framework to work with high-throughput sequencing data. *Bioinformatics (Oxford, England)* 2015; 31: 166-169.

6	Anders S, Huber W. Differential expression analysis for sequence count data. *Genome Biol* (Research Support, Non-U.S. Gov't) 2010; 11: R106.

7	Love MI, Huber W, Anders S. Moderated estimation of fold change and dispersion for RNA-seq data with DESeq2. *Genome Biol* 2014; 15: 550.

8	Szklarczyk D, Gable AL, Lyon D, Junge A, Wyder S, Huerta-Cepas J *et al*. STRING v11: protein-protein association networks with increased coverage, supporting functional discovery in genome-wide experimental datasets. *Nucleic Acids Res* 2019; 47: D607-d613.

9	Subramanian A, Tamayo P, Mootha VK, Mukherjee S, Ebert BL, Gillette MA *et al*. Gene set enrichment analysis: a knowledge-based approach for interpreting genome-wide expression profiles. *Proceedings of the National Academy of Sciences of the United States of America* 2005; 102: 15545-15550.

10	Liberzon A, Birger C, Thorvaldsdóttir H, Ghandi M, Mesirov JP, Tamayo P. The Molecular Signatures Database (MSigDB) hallmark gene set collection. *Cell systems* 2015; 1: 417-425.

11	Li H, Durbin R. Fast and accurate short read alignment with Burrows-Wheeler transform. *Bioinformatics (Oxford, England)* 2009; 25: 1754-1760.

12	Li H, Handsaker B, Wysoker A, Fennell T, Ruan J, Homer N *et al*. The Sequence Alignment/Map format and SAMtools. *Bioinformatics (Oxford, England)* 2009; 25: 2078-2079.

13	Koboldt DC, Larson DE, Chen K, Ding L, Wilson RK. Massively parallel sequencing approaches for characterization of structural variation. *Methods Mol Biol* 2012; 838: 369-384.

14	Wang K, Li M, Hakonarson H. ANNOVAR: functional annotation of genetic variants from high-throughput sequencing data. *Nucleic Acids Res* 2010; 38: e164.

15	O'Leary NA, Wright MW, Brister JR, Ciufo S, Haddad D, McVeigh R *et al*. Reference sequence (RefSeq) database at NCBI: current status, taxonomic expansion, and functional annotation. *Nucleic Acids Res* 2016; 44: D733-745.

16	Sherry ST, Ward MH, Kholodov M, Baker J, Phan L, Smigielski EM *et al*. dbSNP: the NCBI database of genetic variation. *Nucleic Acids Res* 2001; 29: 308-311.

17	Tamborero D, Rubio-Perez C, Deu-Pons J, Schroeder MP, Vivancos A, Rovira A *et al*. Cancer Genome Interpreter annotates the biological and clinical relevance of tumor alterations. *Genome medicine* 2018; 10: 25.

18	Sondka Z, Bamford S, Cole CG, Ward SA, Dunham I, Forbes SA. The COSMIC Cancer Gene Census: describing genetic dysfunction across all human cancers. *Nature reviews Cancer* 2018; 18: 696-705.

19	Tate JG, Bamford S, Jubb HC, Sondka Z, Beare DM, Bindal N *et al*. COSMIC: the Catalogue Of Somatic Mutations In Cancer. *Nucleic Acids Res* 2019; 47: D941-d947.

20	Boeva V, Popova T, Bleakley K, Chiche P, Cappo J, Schleiermacher G *et al*. Control-FREEC: a tool for assessing copy number and allelic content using next-generation sequencing data. *Bioinformatics (Oxford, England)* 2012; 28: 423-425.

**SUPPLEMENTARY TABLES**

**Supplementary Table S1.** Single nucleotide variants (<200 bp) in primary cells derived from the spleens of sick preleukaemic (*TgSpi1FA^+/+^*) and leukaemic (*TgSpi1FA^-/-^*) mice were identified by exome sequencing and called as described in the methods. Mouse identification number is indicated.

The official name corresponds to the accession name of RNA corresponding to the genes (NCBI). Variant allele frequencies (VAFs) are indicated.

**Supplementary Table S2.** Copy number variation (>200 bp) in primary cells derived from the spleens of sick preleukaemic (*TgSpi1FA^+/+^*) and leukaemic (*TgSpi1FA^-/-^*) mice were identified by exome sequencing and called using Control-FREEC. Mouse identification number of mice is indicated. Genes located in X and Y chromosome were excluded. For each gene, gain or loss or gain/loss or NA (no gain or loss) status are indicated.

**Supplementary Table S3.** Copy number variation (>200 bp) in primary cells derived from spleens of sick preleukaemic (*TgSpi1FA^+/+^*) and leukaemic (*TgSpi1FA^-/-^*) mice were identified by exome sequencing and called using Control-FREEC. All CNVs are reported, and the positions are indicated for each mouse.

**Supplementary Table S4.** Differential gene expression analysis between *TgSpi1FA^-/-^* leukaemic (3 independent mice, #26, #28, #39) and *TgSpi1FA^-/-^* preleukaemic (3 independent mice, #77, #223, #40) cells cultured in the presence of EPO. Linear FC; linear fold change of leukaemic *TgSpi1FA^-/-^* versus preleukaemic *TgSpi1FA^-/-^*.

**Supplementary Table S5.** Differential gene expression analysis between *TgSpi1FA^-/-^* preleukaemic (3 independent mice, #77, #223, #40) and *TgSpi1FA^+/+^* preleukaemic (4 independent mice, #138, #445, #451, #479) cells cultured in the presence of EPO. Linear FC; linear fold change of preleukaemic *TgSpi1FA^-/-^* versus preleukaemic *TgSpi1FA^+/+^*.

**Supplementary Table S6.** Differential gene expression analysis between *TgSpi1FA^+/+^* leukaemic (3 independent mice) and *TgSpi1FA^+/+^* preleukaemic (3 independent mice) cells cultured in the presence of EPO (model 2). Linear FC; linear fold change of leukaemic versus preleukaemic cells.

**Supplementary Table S7.** Venn diagram depicting the overlap of DEGs retrieved from leukaemic and preleukaemic cells in *TgSpi1FA^-/-^* (model 1) and in*TgSpi1FA^+/+^* (model 2) mice*.* Lists of the common activated or repressed genes are indicated.

**SUPPLEMENTARY FIGURES**

**Supplementary Fig. S1: SPI1 and *Fanca* expression analysis in *TgSpi1FA^-/-^* mice.**

**(A)** *Fanca* mRNA levels in whole bone marrow cells from 3- to 4-month-old *TgSpi1FA^+/+^* (N=4), *TgSpi1FA^-/-^* (N= 5), *FA^+/+^* (N=2) and *FA^-/-^* (N=4) mice were quantified by RT‒qPCR and normalized to the *Polr2a* mRNA level; Left panel: Bars represent the mean±SEM of 2^-ΔCt^ (ΔCt=Ctgene-CtPolr2a) of independent mice. Statistical analysis of the 2^-ΔCt^ values was carried out using unpaired Student’s t-test. ***P<0.001 and *P<0.05. For easier interpretation, the right side of the histograms shows the mRNA expression relative to *FA^+/+^* WT mice. **(B)** Representative images of immunohistochemical staining of SPI1 (brown staining) in bone marrow (upper panels) or spleen (lower panels) sections of 3- to 4-month-old *FA^+/+^*, *FA^-/-^*, *TgSpi1FA^+/+^* and *TgSpi1FA^-/-^* mice. Cell nuclei were counterstained with haematoxylin (blue staining). **(C)** Mortality rate of *FA^+/+^*, *FA^-/-^*, *TgSpi1FA^+/+^, TgSpi1FA^+/+^* and *TgSpi1FA^-/-^* mice at 320 days after birth. Data indicate the number and percentage of dead animals in each group.

**Supplementary Fig. S2: Haematological features of animals.** Scatter dot plots represent the percentage of populations enriched in CD4^+^ T lymphocytes (CD4^+^CD8^-^) **(A)**, CD8^+^ T lymphocytes (CD4^-^CD8^+^) **(B)**, myeloid (CD11b^+^) **(C)** and B lymphocytes (CD19^+^B220^+^) anayzed by flow cytometry, **(D)** from whole bone marrow of from 3- to 4-month-old *FA^+/+^* and *FA^-/-^* mice and moribund *TgSpi1FA^-/-^* and *TgSpi1FA^+/+^* mice. Histograms indicated the mean±SEM. Statistical analysis was carried out using the Mann‒Whitney test; **P*<0.05, ****P*<0.001.

**Supplementary Fig. S3: The sole *Fanca* deletion is not sufficient for EPO independence. (A)** Images of *in situ* tumours after secondary subcutaneous transplantation into nude mice. **(B)** The percentage of proliferating CFU-E blasts (CD71^+^, Ter119^-^, IL3R^-^, KIT^+^) in bone marrow or spleen of sublethally irradiated NSG mice intraveinously transplanted with 0.5x10^6^ EPO dependent (Dep.) preleukaemic cells of *TgSpi1FA^+/+^* (#445, #479) or EPO independent (Ind.) leukaemic cells of *TgSpi1FA^-/-^* (#26, #28, #39) mice. **(C)** RT-qPCR of *Fanca* mRNA in *TgSpi1FA^+/+^* preleukaemic cells derived from three independent sick mice 7 days postinfection with virus expressing shRNA against *Fanca* (sh*FA*) or scrambled shRNA (shCTRL) (mice #445, #479, #138). Histograms represent the mean expression of two experiments relative to their respective control shRNA ±SEM. **(D)** Preleukaemic cells isolated from the spleens of sick *TgSpi1FA^+/+^* mice infected with shCTRL (blue line) or sh*Fanca* (shFA, red line) were seeded 7 days postinfection at 5x10^4^ cells/mL in liquid medium with (+EPO, full line) or without EPO (-EPO, dashed line). Living cells were counted on the days indicated using the trypan blue exclusion test. Data are the mean±SEM of 3 independent experiments.

**Supplementary Fig. S4: Analysis of Fanconi anemia hallmarks in SPI1 overexpressing cells. (A)** Frequencies of aberrations per metaphase (chromosomal breaks, gaps, fusions, and large deletions) scored in ≥50 metaphases per point (n = 3, * *P*<0.05, Student’s t-test). **(B)** Representative images of TgSpi1FA^+/+^ and TgSpi1FA^-/-^ cells stained for 53BP1 (left) and percentages of 53BP1-positive cells (right; n = 3, **P*<0.05, Student’s t test, ≥100 cells analysed per point). The means±SEM are shown. **(C)** Relative intracellular level of reactive oxygen species (ROS) measured with the DCF-DA probe via flow cytometry. The data represents the means±SEM of 3 independent experiments. **P*<0.05, Student’s t test.

**Supplementary Fig. S5 :** Comparison of the variant nucleotides identified in preleukaemic *TgSpi1FA^+/+^* and leukaemic *TgSpi1FA^-/-^* cells with those reported in the human COSMIC database.

**Supplementary Fig. S6:** NOTCH and WNT/β-catenin pathways are enriched in FANCA-deficient leukaemic cells. **(A)** Score of the NOTCH or WNT/β-catenin pathways enrichment identified using the GSEA algorithm comparing *TgSpi1FA^-/-^* leukaemic to preleukaemic cells **(B)** Validation using RT-qPCR of mRNA expression of DEG (|FC|≥1.5 & *P*<0.05) belonging to WNT/β-catenin or NOTCH pathways identified by RNA-seq (Fig. 6B); PL: preleukaemic; L: leukaemic. The upper part presents the mean of ΔCt (*Cttarget-Ctgapdh*) ± SEM of three independent mice for which RNA expression was measured twice. For easier interpretation, we presented the histograms in the lower part representing the expression relative to *TgSpi1FA^+/+^* preleukaemic cells from respective model (2^-ΔΔCt^), calculated from the mean of the ΔCt, reason why the SEM are indicated on the ΔCt.
